# Surrogate end points and their role in clinical trials: Reply from authors

**DOI:** 10.4103/0253-7613.48879

**Published:** 2009-02

**Authors:** M.V. Kalikar, V.R. Thawani, U.K. Varadpande, S.D. Sontakke, R.P. Singh, R.K. Khiyani

**Affiliations:** Department of Pharmacology, Government Medical College, Nagpur, India; 1 Department of Preventive and Social Medicine, Government Medical College, Nagpur, India; 2 Department of Skin and Venereal Diseases, Government Medical College, Nagpur, India; 3 Department of Rasa Shastra, Government Ayurvedic College, Nagpur, India. E-mail: vijaythawani@rediffmail.com

Sir,

We agree that surrogate markers should meet the requirements of easy availability and affordability, and that the end points should be reliable, exhibit a dose response effect, and be quantifiable and reproducible.[1]

It was precisely for this reason that in our study,[2] CD4 was used as a marker, which is universally accepted, claimed to be reliable, a true predictor, specific and sensitive for predicting the outcome and progression of HIV infection. The CD4 count is also used to assess therapeutic efficacy. However, it has no precise cut off point.

Since it has not been documented whether the immunity offered by tinospora is cell mediated or humoral, other *in vitro* methods were not used by us. Hence, we complemented our study with clinical monitoring as well, for the evaluation of the therapeutic effect of tinospora.

The other specific marker used in measuring the therapeutic effect in HIV positive patients is the viral load. However, as quoted in the first point by the learned author, this was not available in our institution at the time this study was commissioned and due to financial constraints, it could not be procured from elsewhere. Our study was not industry sponsored and was funded by an NGO, without any conflict of interest.

In a research protocol, a hypothesis is formulated and after approval by the EC (Ethical Committee), the application for grant is made. At the planning stage, we expected that CD4 count may give us some correlation with therapeutic effect; however, at the end of study, on evaluation, it was not found to be so. It may be appreciated that our findings truthfully report the poor correlation of CD4 count with clinical findings with six months' use of *Tinospora cordifolia* extract in human immuno deficiency virus positive patients.
